# Targeting Extracellular Vesicles to Dendritic Cells and Macrophages

**DOI:** 10.32607/actanaturae.11478

**Published:** 2021

**Authors:** L. A. Ovchinnikova, I. N. Filimonova, M. Y. Zakharova, D. S. Balabashin, T. K. Aliev, Y. A. Lomakin, A. G. Gabibov

**Affiliations:** Shemyakin-Ovchinnikov Institute of Bioorganic Chemistry RAS, Moscow, 117997 Russia; Pirogov Russian National Research Medical University, Moscow,117997 Russia; Lomonosov Moscow State University, Moscow,119991 Russia

**Keywords:** extracellular vesicles, nanocages, APC, targeted delivery, nanobody, CD206, VSV-G

## Abstract

Targeting protein therapeutics to specific cells and tissues is a major challenge in modern medicine. Improving the specificity of protein therapeutic delivery will significantly enhance efficiency in drug development. One of the promising tools for protein delivery is extracellular vesicles (EVs) that are enveloped by a complex lipid bilayer. EVs are secreted by almost all cell types and possess significant advantages: biocompatibility, stability, and the ability to penetrate the blood–brain barrier. Overexpression of the vesicular stomatitis virus protein G (VSV-G) was shown to promote EV formation by the producer cell. We have developed an EV-based system for targeted delivery of protein cargoes to antigen-presenting cells (APCs). In this study, we show that attachment of a recombinant llama nanobody α-CD206 to the N-terminus of a truncated VSV-G increases the selectivity of EV cargo delivery mainly to APCs. These results highlight the outstanding technological and biomedical potential of EV-based delivery systems for correcting the immune response in patients with autoimmune, viral, and oncological diseases.

## INTRODUCTION

The current rapid progress in modern biomedicine is based on the development of therapeutic drugs with high selectivity and low toxicity. The design of these drugs is associated with the development of highly active therapeutic components and also with their effective delivery to certain organs, tissues, and target cells [1, 2]. The current significant progress in targeted drug delivery has been achieved using antibody targeted therapy, darpins, and nanoparticles [3, 4, 5, 6]. The use of extracellular vesicles (EVs) as carriers of protein molecules has a number of advantages: (1) natural biocompatibility of the cell membrane and EV membranes; (2) the ability of EVs to penetrate the blood–brain barrier; and (3) the possibility of changing the protein composition of the EV membrane [7]. Modification of the protein profile of EV membranes enables a targeted delivery of therapeutic EV cargoes into the desired cells [8, 9].

The precursors of EVs in the targeted delivery of therapeutic drugs and the most extensively studied carriers are liposomes. Many liposome-based drugs have successfully passed clinical trials and been introduced into clinical practice [10, 11, 12]. One of the promising liposome-based agents for the treatment of multiple sclerosis (MS) is Xemys [13, 14, 15]. This agent consists of mannosylated liposomes loaded with immunodominant peptides of the myelin basic protein (MBP). Therapeutic peptides are delivered directly to antigen-presenting cells (APCs) – dendritic cells (DCs) and macrophages (MPs) – by means of the mannose residues on the liposome surface. The presumptive mechanism of action is hyperpresentation of the delivered MBP fragments by the class II major histocompatibility complex on the APC surface, which causes immunosuppression and suppression of autoimmune inflammation. This agent has successfully passed preclinical trials and phase II clinical trials. Phase III clinical trials are expected to be carried out prior to approval for use in the Russian Federation. However, the treatment of MS requires a regular, lifelong administration of these liposomes to the patient, which is associated with economic costs and inconvenience for patients. EVs may be more convenient carriers of MBP fragments for the long-term therapy of MS patients. The existing methods for EV production [16] enable the development of genetically encoded EVs loaded with MBP peptides. The use of autologous human cells as producer cells will provide a transition towards personalized medicine and avoid the need for regular injections that reduce the quality of life [17].

This paper describes a system for the targeted delivery of the EV content to APCs. A DC and MP surface marker, CD206 (mannose receptor), was chosen [18], by analogy with Xemys. This receptor binds glycoconjugates terminated in mannose, fucose, or N-acetyl-dglucosamine residues, which are abundantly present on the surface of pathogenic microorganisms [19]. Conformational changes in the receptor, which are induced by interaction with a mannose residue, lead to the internalization of the bound pathogen and its transport to lysosomes [20], which explains the high expression level of this receptor on DCs and MPs–classical APCs of the human immune system. We have developed a system for the production of EVs with a surface-displayed llama nanobody specific to human and mouse CD206. These vesicles are about 100–140 nm in size and carry exosomal markers [7]. We have shown the possibility of delivering a cargo protein to the desired cells, including human DC and MP, using targeted vesicles. The obtained data will enable the use of the strategy of targeting genetically encoded vesicles to APCs for the development of agents to correct the immune response in patients with autoimmune, viral, and oncological diseases.

## EXPERIMENTAL PROCEDURES

**Plasmids**

To produce the pCMV-NanoLuc-Jun construct (Addgene ID: 167308), the gene encoding NanoLuc luciferase was amplified from the For_NanoLuc and Rev_NanoLuc primers (*Table*) and ligated into the pCMV-Jun vector at the *HindIII/KpnI* restriction sites. The sequence encoding a truncated VSV-G (pCMV-VSV-G_truncated) (amino acid sequence:

**Table:** Primers used to generate the constructs

Primer	Sequence
For_CD206	5’-TGGGGTGAATTGCTTCGGAAGTCAGGTTCAACTGCAGGAGTC-3’
Rev_CD206	5’-GAATGTGAGGATGTTCGAAGCTGCCTCCTCCTGAGC-3’
For_NanoLuc	5’-TCTGGTACCATGGTCTTCACACTCGAA-3’
Rev_NanoLuc	5’-GGGTGGTGGTGGTGGCAAGCTT-3’
For_VSVG_trunc	5’-GGGGTGAATTGCTTCGAACATCCTCACATTCAAG-3’
Rev_VSVG_trunc	5’-AGAGATGAACCGACTTGGAAAGGGCTCC-3’

All cell lines were maintained at 37°C and 8% CO_2_.

The gene encoding the llama nanobody α-CD206 (clone 3.49) [21] was synthesized and cloned at the 5’-end of the truncated VSV-G into the pCMV-VSVG_ truncated construct for eukaryotic expression and into the pET22 vector for prokaryotic expression. For the production of the recombinant llama antibody α-CD206 in a prokaryotic expression system, a histidine tag for affinity purification and a 3xFLAG epitope for detection with secondary antibodies were added to the protein C-terminus.

**Cell lines**

HEK293T cells were cultured in a complete DMEM medium supplemented with 10% fetal bovine serum (Gibco, USA); Jurkat and DC2.4 cell lines were cultured in a complete RPMI medium supplemented with 10% fetal bovine serum (Gibco, USA).

To produce stimulated DC and MP populations, mononuclear cells (MNCs) were isolated from human peripheral blood by centrifugation in a Ficoll gradient. The resulting cells were incubated in a complete RPMI medium supplemented with 10% fetal bovine serum until the DC and MP precursors adhered to the plastic. Thereafter, non-adherent cells were removed and IL-4 (50 ng/mL) and GM-CSF (100 ng/mL) were added to the adherent cells. Differentiation of MNCs into dendritic cells was performed for 6 days with a change of medium containing a fresh portion of cytokines every 2 days.

**Production of the llama antibody α-CD206- FLAG in a prokaryotic expression system**

The recombinant llama antibody α-CD206 was produced in a prokaryotic expression system, *E. coli *BL21 (DE3) cells. An overnight cell culture was inoculated into a 2xYT medium at a 1:100 ratio and grown to OD_600_ = 0.6. Expression was induced by the addition of 1 mM isopropyl-β-D-1-thiogalactopyranoside. The culture was incubated under high aeration at 28°C for 16 h. Then, it was centrifuged at 3,500g and 4°C for 10 min. The resulting pellet was resuspended in lysis buffer (50 mM Tris-HCl pH 8.0; 150 mM NaCl; 1 mM PMSF) and added with lysozyme to a final concentration of 0.2 mg/mL. Cells were incubated at room temperature until the solution became viscous. The cell mass was disintegrated ultrasonically. The resulting solution was centrifuged at 20,000 g and 4°C for 10 min. The supernatant was filtered through a 0.45 μm filter and loaded onto a Ni-NTA column (Qiagen). Impurity proteins were removed by washing the column with the loading buffer (50 mM Tris-HCl pH 8.0; 150 mM NaCl) and wash buffer with imidazole (50 mM Tris-HCl pH 8.0; 150 mM NaCl; 20 mM imidazole). The antibody was eluted by buffer (50 mM Tris-HCl pH 8.0; 150 mM NaCl; 350 mM imidazole).

**Staining of DCs and MPs with the recombinant llama antibody α-CD206**

The possibility of using the recombinant llama antibody α-CD206 for the targeted delivery of protein therapeutics to APCs was verified in DCs and MPs from human peripheral blood. For this purpose, 500,000 cells were washed twice in PBS buffer, re-suspended in 100 μL of a solution containing 15–00 μg/mL of the recombinant llama antibody α-CD206-FLAG, and incubated at 4°C and constant gentle stirring for 1 h. After incubation, the cells were washed twice with PBS and stained with an anti-FLAG epitope secondary antibody conjugated with a fluorescent PE label according to the manufacturer’s protocol (BioLegend, USA). For control staining, a PE anti-human CD206 antibody (BioLegend, USA) was used. As a negative control, HEK293T cells and non-stimulated MNCs were stained.

**Production and purification of extracellular vesicles**

EVs were produced in HEK293T cells. For this purpose, the cells were concomitantly transfected with 3 constructs: pCMV-VSV-G (or pCMV-VSV-G_truncated, or pCMV-α-CD206_VSV-G_truncated), pCMV-EPN, and pCMV-NanoLuc after reaching 90% confluence. The EV-containing cell medium was harvested after 48 h and subjected to differential centrifugation (300 g for 10 min and 1,000 g for 20 min). The supernatant was filtered through a 0.4 μm membrane and concentrated using Amicon Ultra-0.5 mL 10 kDa centrifugal filters (Millipore, Ireland). The concentrate was washed several times with PBS to remove off-target proteins. The EV concentration was determined using a CBQCA Protein Quantitation Kit (Invitrogen, USA).

**Incubation of extracellular vesicles with cells**

EVs carrying the reporter protein luciferase were aligned according to the protein concentration in the sample, added to 300,000 cells (Jurkat and DC2.4), and incubated at 37°C and 8% CO_2_ for 2 h. Soluble Nano- Luc-Jun luciferase, not loaded into EVs, was used as a control. After incubation, the cells were washed with PBS at 300 g for 10 min and incubated in buffer with proteinase K (Invitrogen, USA) to a final concentration of 0.1 mg/mL at 37°C for 15 min. After incubation, the cells were washed twice in PBS. The NanoGlo Luciferase Assay System (Promega, USA) was used to analyze the luciferase content in the cells. For the assay, 30,000 cells were resuspended in 15 μL of PBS and added to 15 μL of the lysis buffer containing a luciferase substrate. The signal was detected on a Varioskan plate reader (Thermo Scientific, USA) at 460 nm.

**Targeted delivery of NanoLuc to DCs and MPs using targeted EVs**

A heterogeneous population of stimulated DCs and MPs from human peripheral blood was added with targeted EVs (carrying the truncated VSV-G variant fused with the α-CD206 antibody on their surface) at a concentration of 5–20 μg/mL and incubated at 37°C and 8% CO_2_ for 2 h. The cells were then gently washed according to the above-described procedure, re-suspended in the complete DMEM medium, and incubated in a vesicle-free medium for 16 h. After 16 h, the cells were stained with a PE anti-human α-CD206 antibody (Biolegend, USA). The cells were sorted on a Sony SH800 cell sorter (Germany). Two cell subsets, CD206^+^ and CD206^-^, were sorted. For the luciferase assay, 30,000 cells were taken from each subset.

## RESULTS AND DISCUSSION

**Production of a recombinant antibody specific to the surface marker of dendritic cells and macrophages**

For the targeted delivery of EVs cargoes to APCs, we chose the DC and MP (M2) surface marker CD206 (macrophage mannose receptor) [18]. We selected the cross-reactive llama nanobody Nb3.49 interacting with the human and mouse mannose receptor [21]. This cross-reactivity is extremely useful in preclinical studies of targeted extracellular vesicles in mouse models, while this antibody can be also used in clinical trials. To test the functionality and specificity of this antibody, we created the recombinant nanobody α-CD206-FLAG in a prokaryotic expression system, based on the pET22 vector. A histidine tag was used for detection and affinity purification; additionally, a 3xFLAG epitope was fused to the N-terminus of the protein to increase the detection sensitivity.

**Fig. 1:**
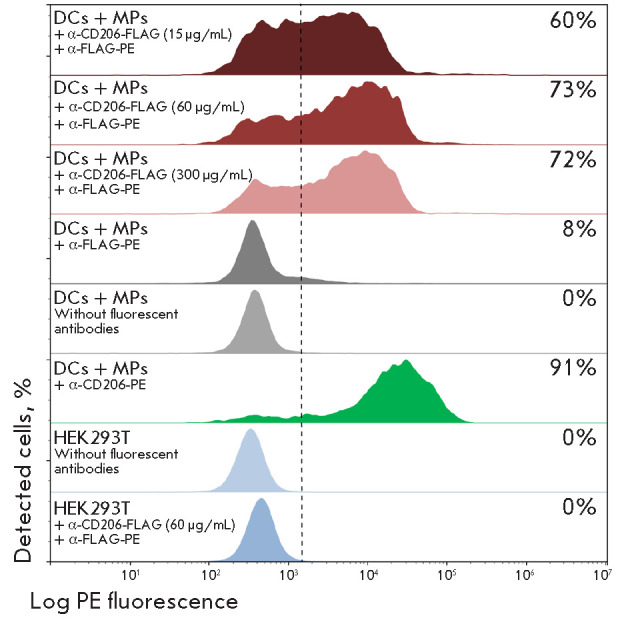
FACS analysis of DC and MP staining with the recombinant α-CD206 nanobody. DC and MP differentiation from human peripheral blood MNCs was stimulated by using IL-4 and GM-CSF for 7 days. Cell binding with the recombinant nanobody α-CD206-FLAG was visualized with a fluorescent secondary antibody, α-FLAG-PE (dark red, red, pink), or a commercially available antibody, α-CD206-PE (green). Unstained cells and cells stained with secondary antibodies alone (α-FLAG-PE) are shown in grey. The lower panel shows control binding of the recombinant nanobody α-CD206-FLAG with HEK293T cells (blue). The X axis shows the fluorescence signal intensity, and the Y axis shows the number of positive events. Each histogram shows the percentage of cells bound to the analyzed antibodies

The specificity of the produced nanobody was verified in a subset of human DCs. For this purpose, mononuclear cells (MNCs) from human peripheral blood were cultured in a complete culture medium in the presence of IL-4 and GM-CSF for a week, with partial replacement of the medium every two days. Under these conditions, the differentiation of DC and MP is stimulated in the culture of human lymphocytes [22]. The purified recombinant nanobody α-CD206-FLAG was added to the resulting DC culture, and, then, after incubation and washing, the anti-FLAG epitope secondary antibody conjugated with a fluorescent PE label was added for the detection (*Fig. 1*). Staining of stimulated human MNCs using the recombinant nanobody α-CD206-FLAG enabled clear detection of a DC subset comparable with a subset isolated by staining with the commercially available fluorescent antibody α-CD206-PE. Thus, we had confirmed the functionality and specificity of α-CD206-FLAG in the llama nanobody format. This allows further EVs utilization for targeted protein delivery to APCs.

**Extracellular vesicle content delivery into cells**

Evaluating the effectiveness of a specific delivery of a therapeutic agent into target cells is an essential stage in the development of protein drug carriers. The most convenient way of undertaking this evaluation is to use fluorescent proteins or luciferase as the agent to be delivered. A significant disadvantage of the use of fluorescent proteins for these purposes is their high molecular weights and the need to use highly sensitive detection methods. For this reason, we used NanoLuc luciferase as the agent to be delivered. This luciferase has good spectral characteristics and a small size of 19 kDa.

**Fig. 2:**
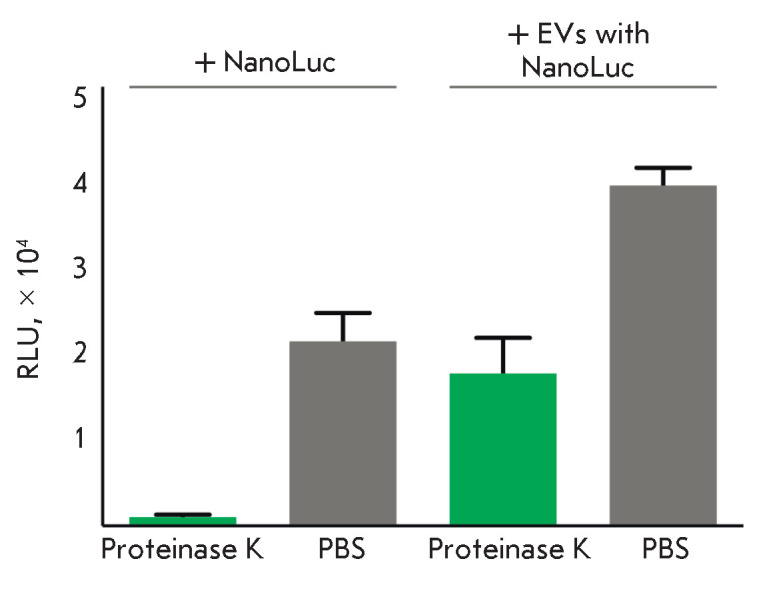
Detection of NanoLuc delivered to target cells via EVs. An additional step of incubation with proteinase K (green bars) enables detection of the luciferase delivered into the cells without a nonspecific signal from NanoLuc adhered to the cell membrane. Cells washed with PBS alone are shown as grey bars. A luciferase assay was used to detect NanoLuc delivered inside the cells

The surface of target cells is covered with a large amount of membrane proteins. These proteins are able to mediate a nonspecific interaction of soluble proteins with target cells *in vitro*, distorting the visualization of the real distribution of delivered EVs’ cargoes among cells. In our experiments, we minimized the level of the nonspecific signal mediated by the adhesion of soluble (not encapsulated in vesicles) luciferase by additional incubation of cells with proteinase K. Extracellular vesicles loaded with luciferase and soluble NanoLuc were added to the target cells. After incubation for 2 h, the cells were washed free of the vesicles and soluble NanoLuc with phosphate-buffered saline alone or with further incubation with proteinase K. As can be seen from *Fig. 2*, the incubation of cells with proteinase K reduces the non-specific signal level compared to that in cells incubated in buffer without proteinase K. In this case, the signal from cells incubated with EVs is more than an order of magnitude higher than that from cells incubated with soluble NanoLuc. The use of proteinase K in the washing steps confirms that the luciferase is delivered into the cells and does not adhere to the membrane. Therefore, we were able to ensure delivery of luciferase into cells using extracellular vesicles and to optimize the conditions for the detection of this signal.

**Fig. 3:**
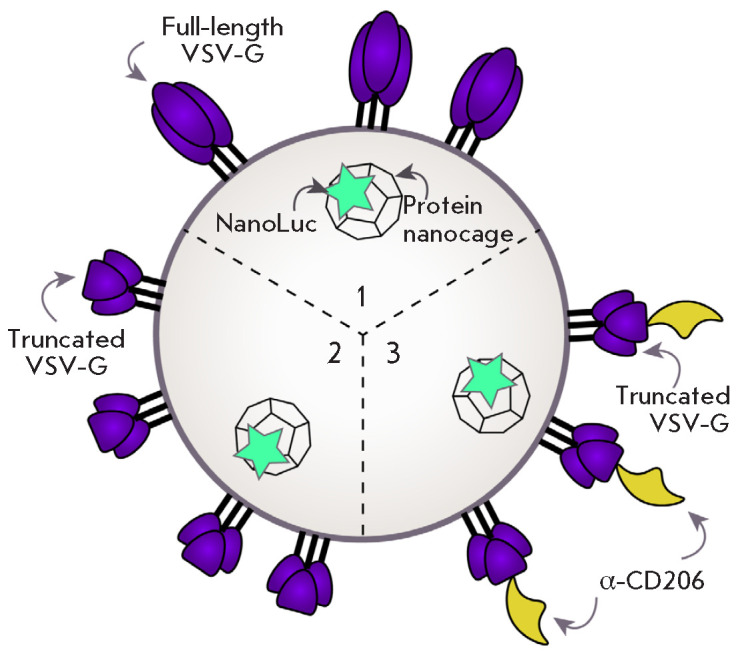
Schematic of an EV with different VSV-G molecules. 1 – full-length VSV-G, 2 – truncated VSV-G, 3 – truncated VSV-G fused with the α-CD206 nanobody

The main component underlying the ability of extracellular vesicles to penetrate into the target cell is the viral glycoprotein VSV-G. This glycoprotein binds to the low-density lipoprotein receptor abundantly present on the surface of mammalian cells [23]. Therefore, using the full-length VSV-G for vesicle content delivery into target cells cannot provide a high specificity of targeted delivery. In our study, we enhanced the specificity of targeted delivery by using a truncated VSV-G. This VSV-G variant comprises only the core part of the protein [24], which is responsible for the budding of extracellular vesicles from the producer cell and the release of the vesicle contents inside the target cell. In this case, it is possible to use a truncated VSV-G sequence combined with a recombinant nanobody capable of highly specific interaction with the target cell, without losing the functionality of the resulting extracellular vesicles. To test the efficiency of agent delivery into the cells, we used EVs loaded with NanoLuc luciferase and carrying various VSV-G variants on their surface: (1) full-size VSV-G, (2) truncated VSV-G, and (3) truncated VSV-G with a surface-exposed nanobody that specifically recognizes the dendritic cell and macrophage marker CD206 (*Fig. 3*).

**Fig. 4:**
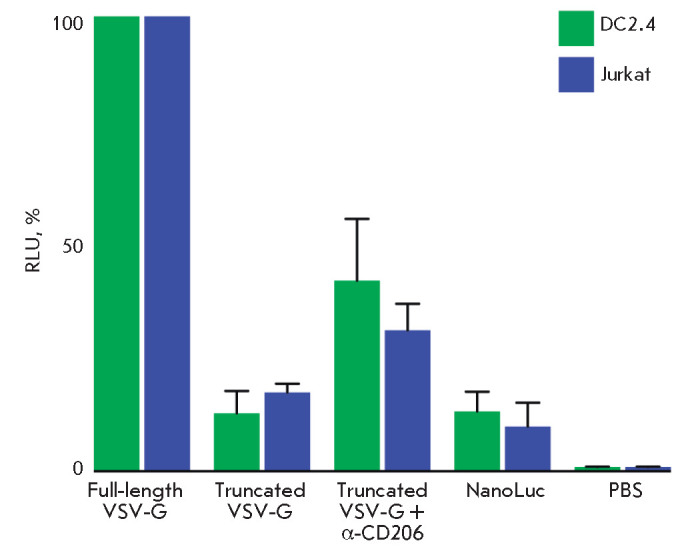
Comparison of protein delivery into target cells using EVs exposing different VSV-G molecules. Delivery analysis was performed in DC2.4 (green bars) and Jurkat (blue bars) cell lines. The delivery efficiency with the full-length VSV-G was taken as 100% for each cell line. Soluble luciferase NanoLuc without vesicles (sample NanoLuc) was used as a control

To test the functioning of vesicles carrying various variants of the VSV-G glycoprotein, we used the DC2.4 mouse dendritic cell line and Jurkat cell line (immortalized human T cells). The cells were incubated with various vesicle variants or a solution of free luciferase and washed in the presence of proteinase K. RLU values obtained in the luciferase assay are shown in *Fig. 4*. In this experiment, the values obtained during the incubation of cells with vesicles carrying the full-length VSV-G were taken as 100%, because, in this case, there was maximum interaction between the vesicles and target cells. The use of a truncated VSV-G reduces the efficiency of luciferase delivery to target cells 5- to 10-fold. This is associated with impaired recognition by the low-density lipoprotein receptor. Fusion of the α-CD206 nanobody with the truncated VSV-G significantly increased the targeted protein delivery to the target cells. In this case, the use of the α-CD206 antibody provided a more efficient delivery of the protein to DC2.4 dendritic cells than to Jurkat cells.

**Fig. 5:**
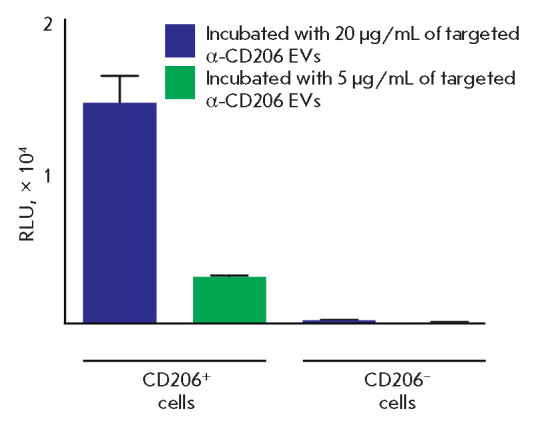
Targeted protein delivery to CD206+ cells using EVs. Targeted α-CD206 EVs loaded with NanoLuc were incubated with stimulated DCs and MPs from human peripheral blood. After sorting of the CD206+ and CD206– subsets, the NanoLuc protein was shown to be delivered predominantly inside CD206+ cells. The same quantity of CD206+ and CD206– cells was analyzed in the luciferase assay

In the future, extracellular vesicles are planned to be used for the targeted delivery of therapeutic agents in the human body. However, the use of immortalized cell lines does not allow for a reliable reconstruction of the actual APC distribution and marker expression level on the cell surface. To prove the functionality of the developed targeted extracellular vesicles loaded with a truncated VSV-G in a heterogeneous cell population, we used human peripheral blood MNCs subjected to stimulated DC and MP differentiation. Targeted extracellular vesicles loaded with luciferase were incubated with a heterogeneous population of CD206+ and CD206– cells. Next, the analyzed cells were washed, stained with the fluorescent antibody α-CD206-PE, and sorted into two subsets of CD206+ and CD206– cells using flow cytometry. The content of luciferase delivered into the target cells was detected separately in the CD206+ and CD206– cell subsets. We were able to achieve a high specificity of luciferase delivery mainly to CD206+ cells (*Fig. 5*).

## CONCLUSIONS

Currently, one of the priorities in drug development is enhancing the selectivity of delivery. In this study, we proposed an improved method for the targeted delivery of protein therapeutics encapsulated in EVs. The high biocompatibility and biodegradability of EVs confers them a huge advantage over artificial nanoparticles. Attachment of the recombinant llama antibody α-CD206 to the N-terminus of a truncated VSV-G increases the selectivity of EV delivery predominantly to CD206+ cells without a significant decrease in the production of these EVs. The functionality of the developed constructs was confirmed in immortalized mouse DC2.4 dendritic cells and heterogeneous subsets of stimulated DCs and MPs from human peripheral blood. On the basis of our findings, the strategy of targeting genetically encoded extracellular vesicles to APCs may be used in the development of drugs for the correction of the immune response in patients with autoimmune, viral, and oncological diseases. Vesicles can deliver not only target proteins, but also lipids, nucleic acids, and transcription factors to cells [1]. In the future, EV-based targeted drug delivery could be used in gene therapy. Currently, many studies focus on the development of EV-based delivery systems. These vesicles are specifically loaded with proteins [25], peptides [26], and RNAs [27, 28]. In this case, there is a serious problem having to do with the transfer of various off-target ballast molecules by the produced EVs. Delivery of undesirable components into the target cell can seriously affect the biocompatibility of the drug and lead to unpredictable side effects. One of the ways to solve this problem is to use autologous cells for the production of vesicles [29]. The safety of these EVs has been confirmed by clinical trials [30, 31, 32]. However, the long-term effect of natural EV content delivery into cells should also be carefully evaluated during the development of potential drugs.

## Abbreviations

APC: antigen-presenting cell
EV: extracellular vesicle
DC: dendritic cell
MP: macrophage
MNC: mononuclear cell
MBP: myelin basic protein
MS: multiple sclerosis
GM-CSF: granulocyte-macrophage colony-stimulating factor
IL: interleukin
VSV-G: vesicular stomatitis virus glycoprotein
